# Deciphering the maize gene ZmGF14–3: implications for plant height based on co-expression networks

**DOI:** 10.3389/fpls.2024.1397058

**Published:** 2024-07-05

**Authors:** Hengsheng Wang, Bo Wei, Lulu Qi, Yansong Chen, Kelong Chen, Dong Liu, Xu Su, Yan Zhang, Lingling Li

**Affiliations:** ^1^ School of Biological and Food Engineering, Hefei Normal University, Hefei, Anhui, China; ^2^ Blueberry Engineering Technology Research Center of Anhui, Hefei Normal University, Hefei, Anhui, China; ^3^ College of Geographic Sciences, Qinghai Normal University, Xining, Qinhai, China; ^4^ School of Biology, Food and Environment, Hefei University, Hefei, Anhui, China; ^5^ Department of Horticulture and Landscape, Anqing Vocational and Technical College, Anqing, Anhui, China; ^6^ Key Laboratory of Biodiversity Formation Mechanism and Comprehensive Utilization of the Qinghai-Tibet Plateau in Qinghai Province, Qinghai Normal University, Xining, Qinhai, China

**Keywords:** plant height, ZmGF14-3, co-expression network, qRT-PCR, endogenous phytohormones

## Abstract

The evolutionary analysis showed that the GF14 family was conserved, however, there was limited evidence linking GF14s to plant height. In our investigations, we discovered a co-expression relationship between ZmGF14s and functionally characterized genes linked to plant height. In the co-expression network, we identified ZmGF14-3, a gene expression exhibiting a positive correlation with plant height in three maize varieties, we postulated that this gene could be intimately linked to plant height development. Subsequently, we cloned ZmGF14-3 from the maize B73 inbred line and overexpressed it in *Arabidopsis*, resulting in markedly dwarfed transgenic phenotypes. Measurements of endogenous phytohormones disclosed a significant reduction in concentrations of Gibberellic Acid 7 (GA_7_) and Indole-3-Acetic Acid (IAA) in the overexpressed *Arabidopsis*, furthermore, qPCR results highlighted a pronounced decrease in the expression levels of plant height-related genes when compared to the wild type, therefore, it is plausible to posit that ZmGF14-3 plays a pivotal role in regulating the growth and development of maize through interactions with various phytohormone-related genes. Thus, delving into the potential interactions between ZmGF14-3 and these genes holds the promise of yielding valuable insights into the molecular mechanisms underpinning plant height development in maize.

## 1 Introduction

In the past few decades, the increase in maize yield per unit area has primarily been attributed to higher planting density, rather than an enhancement in yield potential per plant (Sun et al., 2023). Maize have been adapted for high-density planting, however, high-density planting often makes plants susceptible to lodging, and reduce the yield. To counteract this issue, one cultivation strategy has been to adjust the plant height of maize within a suitable range, plant height is also an important grain yield-associated trait and a close correlation was confirmed between plant height and grain yield in maize (*r^2^* > 0.73) (Adel et al., 2016). Hence, plant height is an important trait for maize breeding.

There were two main biological factors that mainly influence height of maize plant, internode number and internode length, and expand in maize plant height are mainly attributable to increased internode elongation instead of enhance in internode number. Previous studies have established that the regulation of plant height is intricately governed by phytohormones and influenced by a multitude of associated Quantitative Trait Loci (QTLs). Currently, the molecular landscape of plant height in maize has been illuminated by the cloning and characterization of just over 60 genes associated with this trait. These genes span a spectrum of functions, encompassing signaling, transport, and phytohormone synthesis. Notable among them are genes such as *an1*, *br2*, *d1*, *d2*, *d3*, *d5*, *d8*, *d9*, *qPH3.1*, *DWF1*, and *DWF4*, each contributing to the complex regulatory network orchestrating maize plant height (Shang et al., 2020), Specifically, *Nana Plant 1* (*na1*) and *Nana Plant* 2 (*na2*) are distinctively marked by severe dwarfism, signifying their significant contributions to the brassinosteroid (BR) biosynthesis pathway. Their pivotal role in this pathway suggests a potential correlation with reductions in stem internode length (Zhao et al., 2022). *D3*, identified as a maize gene through transposon tagging, plays a crucial role in regulating gibberellin synthesis. The encoded protein exhibits activity at an early stage in the Gibberellin (GA) biosynthesis pathway (Winkler, 1995). *d8* and *d9* are key elements of the GA signal transduction channel that negatively regulate the GA response, which is involved in GA signal transduction pathways via DELLA proteins (Lawit et al., 2010). Genes involved in the expression of polar auxin transport, such as *ZmPIN1a* and *Brachytic2* (*br2*), are also regulated in the genetic control of plant height in maize (Pilu et al., 2007; Li et al., 2018). *ZmACS7* encodes ACC synthase 7 in the ethylene biosynthesis pathway of maize, resulting in dwarf phenotypes in the *zmacs7* mutant. The observed dwarfism emphasizes the pivotal role played by *ZmACS7* in plant growth, deepening our understanding of ethylene synthesis regulation and offering insights into the genetic factors that impact plant architecture (Li et al., 2020).

The GF14 (14–3-3) gene family is widely preserved in eukaryotes, spanning diverse organisms. GF14s proteins, crucial for cellular processes, have a unique structural composition with nine antiparallel α-helices. The persistent structural uniformity in GF14 proteins underscores their significance across various biological contexts. The widespread distribution and conservation of the GF14 genes family emphasize its foundational role in diverse eukaryotic organisms, making it intriguing for further exploration in understanding cellular processes and signaling pathways (Roy et al., 2023). GF14s frequently participate in the creation of heterodimers or homodimers, highlighting their dynamic inclination for molecular associations. Within these dimers, each GF14 protein exhibits an impressive proficiency to interact with distinct partners, unveiling the intricate versatility of GF14 proteins in coordinating diverse cellular interactions (Joshi et al., 2022). Hitherto, GF14–3s have been identified in plants such as rice, tobacco, *Brachypodium distachyon*, soybean, barley, cotton, *Arabidopsis* and maize, but only few have been phylogenetically and functionally characterized (Niti et al., 2017). In recent studies, the significance of GF14s has been increasingly emphasized, particularly in their involvement in carbon and nitrogen metabolism, as well as their crucial roles in responding to both biotic and abiotic stresses. These proteins serve as key regulatory factors within diverse stress signal transduction pathways, influencing the cellular responses to environmental challenges. Furthermore, GF14s play a pivotal role in the signal transduction processes associated with various phytohormones, including but not limited to GA, Brassinosterol (BR), Abscisic acid (ABA), and Ethylene (ET). This multifaceted involvement underscores the broad impact of GF14s in orchestrating intricate signaling networks critical for plant growth, development, and adaptation to changing environmental conditions (Qin et al., 2016). Despite limited evidence linking GF14s to plant height, ongoing investigations are progressively revealing the intricate roles these proteins play in regulating various aspects of plant morphology. This expanding body of research underscores the complexity of GF14 involvement in shaping the overall form and architecture of plants (Liu et al., 2016).

In this research, to further explore the relationship between ZmGF14s and plant height development, we employed weighted gene co-expression network analysis (WGCNA) on a previously published high-throughput RNA-seq dataset. The results showed that ZmGF14s exhibited a significant co-expression pattern with functionally characterized plant height-related genes in maize. Gene expression analysis suggested that only the expression value of ZmGF14–3 increased with the development of maize, indicating that ZmGF14–3 may play a crucial role in plant height regulation. Subsequently, we isolated ZmGF14–3 from the B73 maize inbred line and obtained *Arabidopsis* lines overexpressing ZmGF14–3 with a 35S promoter. Our results demonstrated that overexpression of ZmGF14–3 in *Arabidopsis* led to a reduction in plant height.

## 2 Materials and methods

### 2.1 Maize materials for co-expression network construction and high-throughput transcriptome sequencing data

In order to accurately and comprehensively depict the dynamic changes in gene expression during maize plant height development, in our previous research, three hybrids were chosen, which were defined as high (H, Dan 598×ES40), middle (M, Dan 598 × CML444) and low (L, Dan 598 × FAPW) group, and RNA samples were collected at the jointing stage, big flare period and tasseling stage. Then, RNA-Seq was used to identify and characterize the expression of large quantities of genes, and develop a genome-wide co-expression network providing a gene expression overview for biological process analysis by combining algorithms (Wang et al., 2021). The RNA-seq datasets can available from Gene Expression Omnibus (GEO) in NCBI under accession number GSE115796.

### 2.2 Gene network construction

The weighted gene co-expression network analysis (WGCNA) R package (Version: 4.2.2) was used to construct a gene co-expression network. The expressed genes in the transcriptome data were cluster analyzed, and the soft threshold was calculated using the pick soft threshold function provided by WGCNA. To make the network show an approximate scale-free topology, a power of nine was chosen (model fitting index R^2^ = 0.8). In order to ensure that the network was biologically relevant, the scale-free topology model and the mean connectivity of the network fit were estimated over a series of the soft threshold power β. All the coding sequences were hierarchical clustered by using topological overlap-based dissimilarity measure. Then, the resulting gene dendrogram was used for module detection based on the dynamic tree cut method (minModuleSize = 100 and mergeCutHeight = 0.25). If any two genes were connected in the weighted gene co-expression network, the topology overlap measure provided in WGCNA was used to determine edge weight. The weights ranged from 0 to 1, and reflected the strength of communication between the two genes. Only weights above 0.2 between any two genes were considered to stand for a strong co-expression relationship in our network. Finally, all expressed ZmGF14s and genes related to plant height were used as guide genes for the co-expression network construction, the Cytoscape_v 3.6.1 software was used to display the co-expression network (Hsieh et al., 2023).

### 2.3 RNA isolation and quantitative reverse transcription PCR

The primer Primier 5 software was used to design gene-specific qRT-PCR primers. A Spectrum™ Plant Total RNA Kit (SIGMA, Beijing, China) was used to extract total RNA from *Arabidopsis* stems, all RNA samples were treated with DNase to eliminate any potential trace contaminants of genomic DNA. A reverse transcription kit (Roche) was then used to synthesize first-strand cDNA (templates for qRT-PCR) using 1 mg of RNA, and the cDNA were transcripted by using PrimeScript™ RT Reagent Kit (Takara, Dalian, China). TransStart Top Green qPCR SuperMix kit (TANSGEN, Beijing China) were used for qRT-PCR, and performed in a CFX96 Real Time PCR Detection System (Bio-Rad). The thermal cycling conditions were as follows: an initial denaturation step of 5 min at 95°C, followed by 40 cycles of 10 s at 95°C for denaturation, 15 s at 60°C for annealing and 30 s at 72°C for extension (Wang et al., 2019). The primer pairs employed for qRT-PCR of cloned genes in this study are detailed in **Supplementary Table S1**, with the housekeeping gene ACTIN serving as a designated internal control, and formula 2^-ΔΔCt^ were used for calculating the expression level. The experiment was conducted in triplicate, a described method was used to calculate the relative expression level of the target gene.

### 2.4 Isolation of ZmGF14–3 cDNA

ZmGF14–3 was isolated from a cDNA library of B73 maize inbred line, and cloned into the 1301a vector, which contains the modified 35S promoter of *Escherichia coli* receptors. 5’-GGATCCATGGCTAAGTTTGTT-3’ (Sense) and 5’-CCTAGGTACCGATTCAAACAA-3’ (Antisense), containing the BamHI and PstI cloning sites, were used as primers to amplify ZmGF14–3 cDNA and subcloned in the corresponding sites of the 1301a vector. The 1301a-ZmGF14–3 construct was introduced into Agrobacterium tumefaciens C58C1 by heat shock. The floral-dip method were used in transforming Col-0 *Arabidopsis* plants, transgenic seedlings were selected on kanamycin medium (50 ug mL^-1^). T2 plants that produced 100% kanamycin-resistant plants in the T3 generation were considered homozygous for the selection marker and arranged for further researches. Other experiments were performed using homozygous transgenic plants of T3 generation.

### 2.5 GA, ABA, and IAA quantification

Phytohormone were analyzed by using HPLC-MS/MS system (HPLC ExionLC™ AC; MS Sciex Triple Quadrupole 4500) (Liu et al., 2023). Prior to extraction, samples were fortified with deuterated standards for each compound. Following centrifugation and extraction, the pH of the supernatant was adjusted to 3.0 and partitioned twice with diethyl ether. The organic layers were then consolidated and evaporated using a centrifuge vacuum evaporator. The dry residue was thereafter resuspended in a water: methanol (9:1) solution, filtered, and injected in a HPLC system. The HPLC analytical conditions were as follows: column was HYPERSIL GOLD C18 column (3μm, 2.1mm*100 mm); solvent A was H_2_O (0.1% FA); solvent B was MeOH; gradient program, 90% A from 0 to 0.2 min, 90% A at 3 min and kept to 8min, 10% A at 8.1 min and kept to 10 min; flow rate, 0.3 mL/min; temperature, 35°C; injection volume: 5μL. The mass spectrometer were adopted in negative ionization electrospray mode for different phytohormone measuring. Further method on the determination procedure are given by Jon et al (Jon et al., 2020). Accurate quantification of GA, ABA, and (Indole-3-Acetic Acid) IAA in *A. thaliana* stems was performed by Guocangjian Biotech (targetcrop.com) on UPLC-MS/MS platform.

### 2.6 Subcellular localization analysis

The ZmGF14–3 coding region, excluding the terminator, was cloned and subsequently fused with the subcellular localization vector pCAMBIA1305, incorporating green fluorescent protein (GFP) tags (Abcam, ShangHai, China, ab275766) driven by the CaMV35S promoter. The ClonExpress MultiS One Step Cloning Kit (Vazyme, Najing, C113–01/02) were used to obtain the pCAMBIA1305-ZmGF14–3 vector through homologous recombination method, and the *N. benthamiana* epidermal cells were used as receptor cell for transforming recombinant plasmid pCAMBIA1305-ZmGF14–3 by *Agrobacterium tumefaciformis* infection. Then, the 13-day-old maize B73 etiolated seedlings were used as materials for preparing maize protoplasts. Additionally, the plasmid pCAMBIA1305 with 35S::ZmGF14–3-GFP fusion proteins was transformed to maize protoplasts using the PEG-mediated transformation method and the pCAMBIA1305 vector were also transformed as control, the DAPI (1 µg/mL) staining solution was used to nuclei stain. After incubation in darkness at 22°C for 16 h, the confocal laser scanning microscope (Zeiss LSM 800, Jena, Germany) were used for observing fluorescence signals. The confocal microscope were used to obtain microscopy images and analyzed by using the ZEN 3.1 software (https://www.zeiss.com.cn/microscopy/products/microscope-software/zen.html accessed on 18 September 2022). The DAPI and GFP were detected at excitation of 461 nm and 488 nm (Li et al., 2022).

### 2.7 Transformation of *Arabidopsis*

The coding sequence of ZmGF14–3 was cloned and inserted into the pMD18-T simple vector (TAKARA, Shanghai, China, Code No. 6011) for sequencing. The gene product was then placed under the control of the CAMV35S promoter and subcloned into the pCAMBIA1301a recombinant vector (Abcam, Shanghai, China, ab275753). The construct was introduced into *Agrobacterium tumefaciens* strain LBA4404 by the infection method and transformed into *Arabidopsis thaliana* (Lu et al., 2017).The seeds of transformed *Arabidopsis thaliana* were selected after being broadcast on MS culture plates with spectinomycin (2 mg/ml) and vernalization for 3 days. Resistant seedlings were planted in the soil, the specific primer PCR were used for identification of transformed plants. Their seeds were harvested separately. T3 homozygous seeds were identified by BASTA resistance and used for further experiments.

## 3 Results

### 3.1 Co−expression network of plant height−specific and ZmGF14s

**Figure 1:**
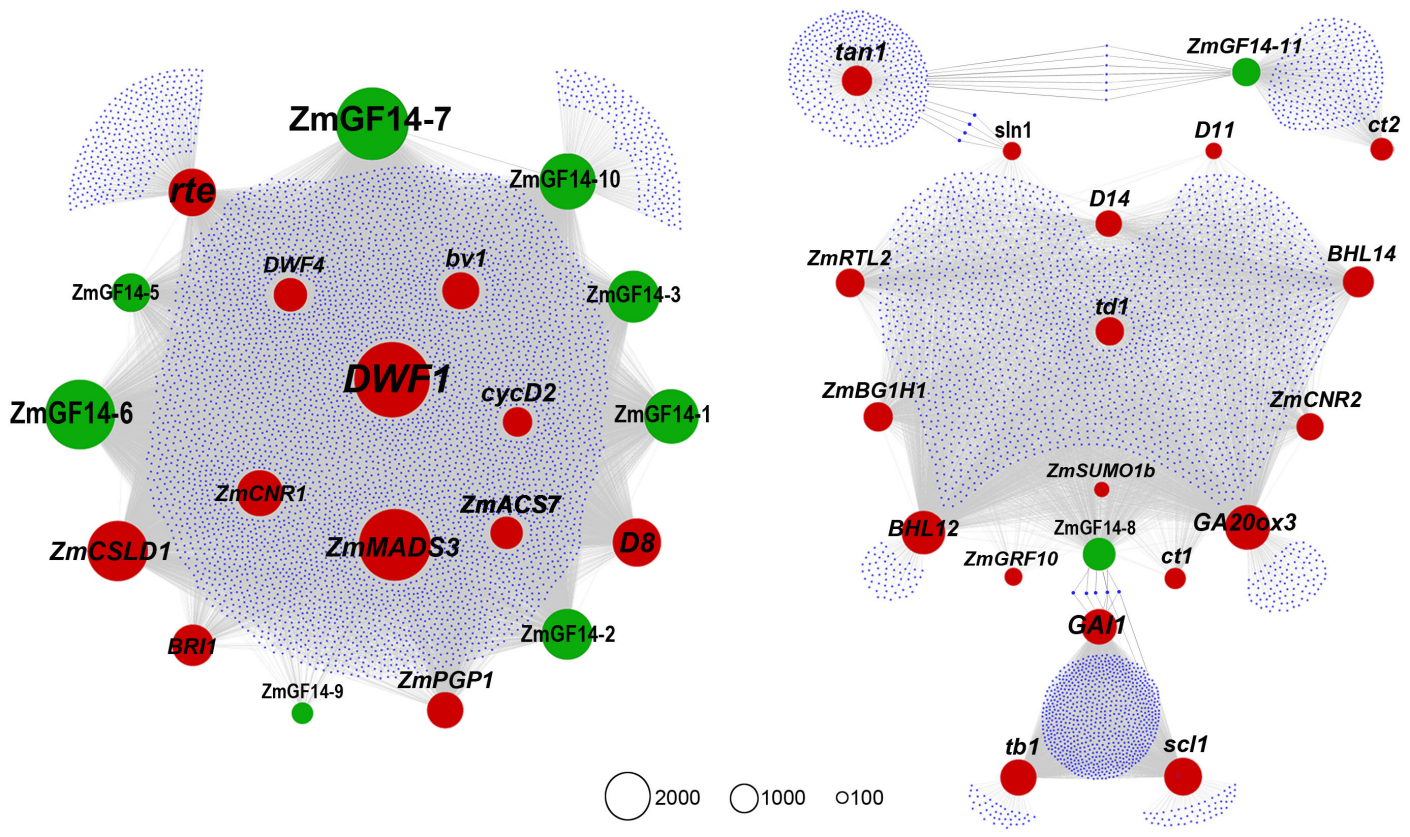
Weighted gene co-expression network of ZmGF14 genes and plant height related genes in maize. Black nodes represent reported plant height-related genes, grey nodes denote ZmGF14 genes, all other genes are depicted as small black nodes. Node size represents total connectivity. An edge indicates significant co-expression between two connected genes.

Currently, a total of 28 ZmGF14s have been reported in maize (Wang et al., 2023). In our previous weighted gene co-expression network analyses (Wang et al., 2018), a total of 12 ZmGF14s were identified (**Supplementary Table S2**). We used these 12 ZmGF14s and all reported maize genes related to plant height as guide genes to construct a co-expression network. As a result, 10 ZmGF14s were identified in the new network, these genes are ZmGF14–1, ZmGF14–2, ZmGF14–3, ZmGF14–5, ZmGF14–6, ZmGF14–7, ZmGF14–8, ZmGF14–9, ZmGF14–10 and ZmGF14–11, and 30 functionally characterized plant height-related genes exhibited a co-expression relationship with these ZmGF14s (**Figure 1**; **Supplementary Tables S3**, **S4**). Among them some key hub ZmGF14s were identified, such as ZmGF14–7 (Co-expressed with 4080 genes), ZmGF14–6 (Co-expressed with 3066 genes), and ZmGF14–3 (Co-expressed with 2028 genes), showed co-expression with *DWF1*, *ZmGRF10*, *rte*, *ZmCNR01*, *D8*, and *bv1*. These results suggest that the 10 ZmGF14s may be potential candidates for regulating plant height.

### 3.2 Phylogenetic analysis of GF14s in different studied species and structural analyses of maize GF14 genes

**Figure 2:**
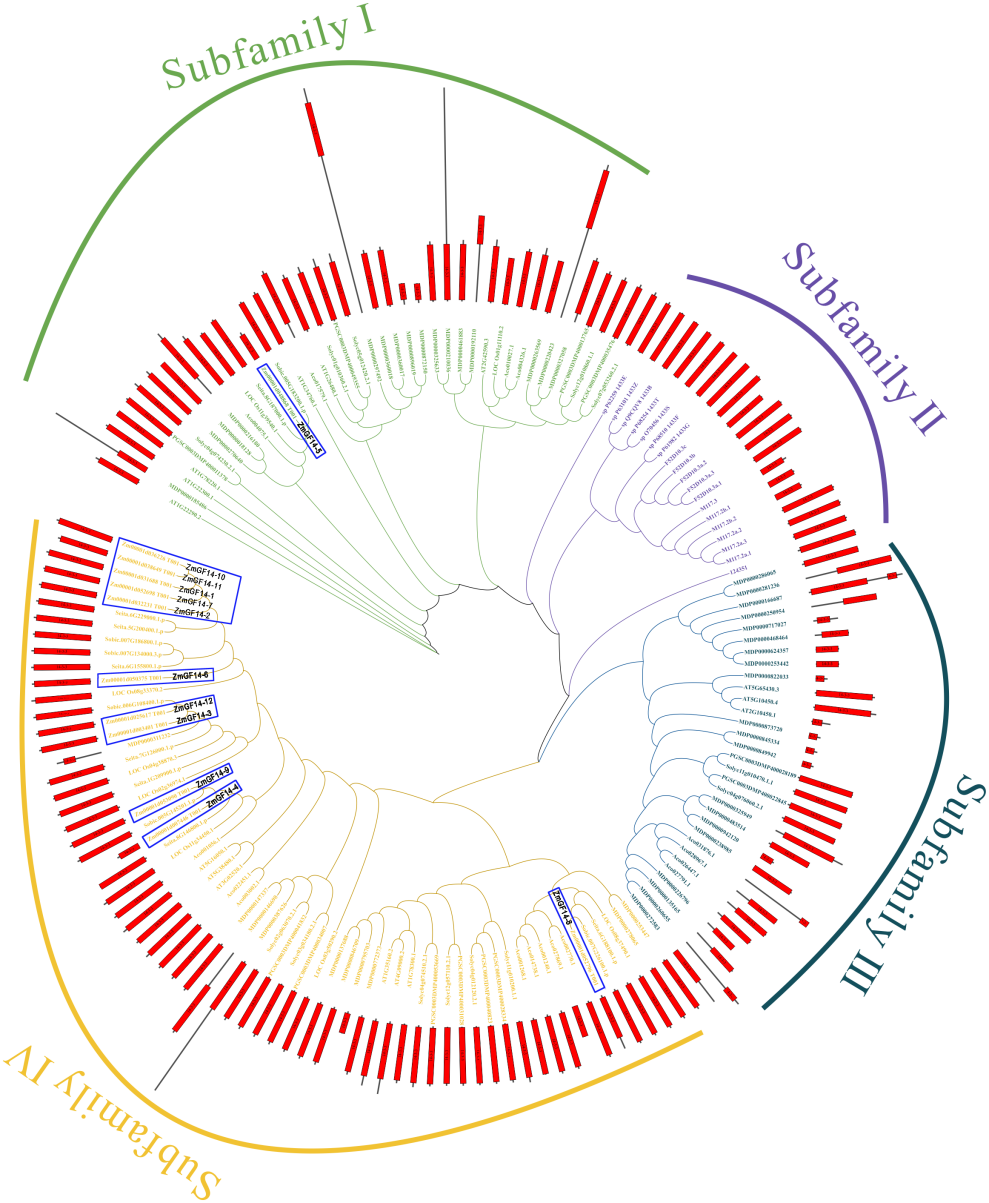
The maximum likelihood evolutionary tree was established for the selected GF14s, which contains 12 selected species and 4 outgroup species and their protein domains. Abbreviation of the species name can be found in **Supplementary Table S5**. The Figure can be divided into four clades, and different clades are shown in different colors. Green, purple, dark green, and orange represent clades I to V, respectively, while the topological structures on the domains are also marked, with red cuboids representing the GF14 (14–3-3) domain.

For a deeper exploration into the evolutionary trajectory of ZmGF14s across maize and other species, we meticulously crafted a maximum likelihood tree encompassing all 155 GF14s identified from 12 meticulously surveyed species (**Figure 2**; **Supplementary Table S5**, **Supplementary File S1**). Based on the topology and bootstrap values of clade nodes, the tree can be categorized into four distinct clades, denoted as Clade I through IV. Within the four clades, 40, 19, 31 and 65 GF14s were clustered, respectively. Clade IV harbored the most GF14s members and Clade II the least, and Clade IV contained GF14s from 8 plant genomes, suggesting the evolutionary conservation and ancient origination of GF14s in these clades. Clade II exclusively comprised GF14s from animals and prokaryotes, suggesting a potentially unique evolutionary trajectory of GF14s across various clades.

We concentrated on GF14s within maize and additionally constructed a neighbor-joining (NJ) phylogenetic tree specifically comprising maize GF14s (**Supplementary Figure S1**). Our findings revealed that the topology of the neighbor-joining phylogenetic tree closely mirrored that of the maximum likelihood phylogenetic tree for GF14s, thereby underscoring the precision of phylogenetic reconstruction. Upon further analysis of maize GF14 gene structures, it was observed that only ZmGF14–5 lacked introns, while the others did not. Additionally, during the investigation of conserved motifs in GF14s, it was discovered that ZmGF14s with close phylogenetic relationships exhibited analogous motif compositions, suggests that these genes may have similar biological functions.

### 3.3 Correlation analysis between ZmGF14s expression and genes associated with plant height

**Figure 3:**
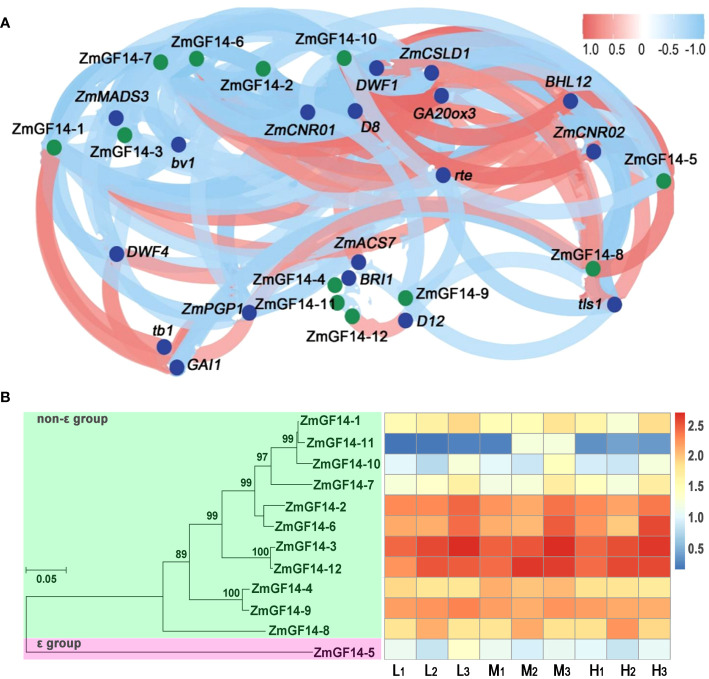
Correlation analysis of gene expression level between ZmGF14s and plant height-related genes and phylogenetic reconstruction. **(A)** Correlation analysis of gene expression level between ZmGF14s and plant height related genes; Blue dots in the figure represent reported height-related genes in maize, while green dots represent ZmGF14 family genes. **(B)** Phylogenetic reconstruction and expression profiles of ZmGFs genes in maize.

To delve deeper into the regulatory interplay between ZmGF14s and genes associated with plant height, we conducted an analysis to evaluate the correlation of gene expression levels across different developmental stages and maize varieties. This assessment was performed through the calculation of the Pearson correlation coefficient. All of the ZmGF14s genes, when compared with functionally characterized plant height-related genes, demonstrated varying degrees of significant correlation (**Figure 3A**; **Supplementary Table S6**). For example, the expression level of ZmGF14–1 exhibited a negative correlation with *GA20ox3* (r = -0.775, *p* -Value = 0.014) and a positive correlation with *DWF1* (r = 0.87, *p*-Value = 0.002). The expression levels of ZmGF14–3 showed positively correlated with *ZmMADS3*, *DWF1*, *D4* and *ZmCNR01* (*p-Value* < 0.05), and negatively correlated with *D11*, *D14*, *ct1* and *BHL14* (*p-*Value < 0.05). Prior investigations have revealed a heightened expression of ZmMADS3 in stem nodes. Ectopically expressing *ZmMADS3* led to reduced plant height, primarily attributed to a decrease in the number of nodes (Heuer et al., 2001). *ZmDWF1* is involved in BRs signal transduction, *zmdwf1* mutant exhibited severity dwarfed phenotype (Tao et al., 2004). *D11* is involved in GA biosynthesis, *d11* mutant exhibited severely developmental abnormalities and showed dwarfed phenotype (Wang et al., 2013). The obtained results posit that ZmGF14s may assume an indirect role within the regulatory framework governing the development of maize plant height. This can be attributed to their extensive involvement across a spectrum of phytohormone metabolic pathways, thereby exerting a discernible influence on the intricacies of plant height modulation.

### 3.4 Expression profiles of ZmGF14s across three developmental stages in three maize hybrids

To gain insights into the evolutionary relationships among maize GF14 proteins, we selectively chose ZmGF14s to construct a neighbor-joining (NJ) phylogenetic tree. The outcomes revealed a distinct classification of all ZmGF14s into two groups: the ϵ group and the non-ϵ group. Furthermore, we examined the relative expression levels of ZmGF14 genes in three maize hybrids (Low: L, Medium: M, High: H) across distinct developmental stages (1: joint stage, 2: trumpet stage, 3: tasseling stage). The analysis unveiled diverse expression patterns among the 12 ZmGF14 genes (**Figure 3B**; **Supplementary Table S7**). These variations in expression patterns imply that specific genes might play crucial roles in the developmental regulation of maize plant height across different growth stages. Notably, ZmGF14–2, 3, 6, and 12 exhibited relatively high expression levels across all hybrids and stages. Specifically, the expression of ZmGF14–3 consistently upregulated throughout the three developmental stages in all three hybrids, suggesting a gradual increase in ZmGF14–3 expression with maize growth and development.

### 3.5 Expression patterns and subcellular localization analysis of ZmGF14–3

**Figure 4:**
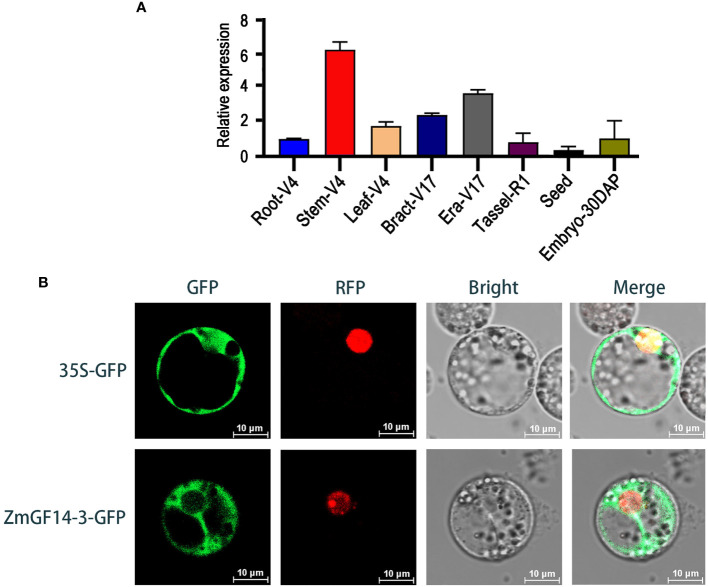
Expression patterns **(A)** and subcellular localization **(B)** analysis of ZmGF14–3.

Building upon the aforementioned findings, we observed that the ZmGF14–3 gene displayed a consistent upward trend in expression across three developmental stages within three maize hybrid lines, each distinguished by varying plant heights categorized as low (L), medium (M), and high (H). Consequently, we infer that ZmGF14–3 may play an important role in the plant height development. Based on these observations, ZmGF14–3 was selected as the focal point for further in-depth functional exploration. In general, gene expression profiles are indicative of their functions (Heyduk et al., 2022). To delve into the expression patterns of ZmGF14–3 throughout maize development, we gathered data on gene expression across nine distinct maize tissues using qRT-PCR. The results revealed that ZmGF14–3 displayed elevated expression levels in the stem (**Figure 4A**), aligning with the data obtained from our previously published transcriptome database.

To ascertain the subcellular localizations of ZmGF14–3, ZmGF14–3-GFP plasmids were introduced into maize protoplasts, with the empty protein (35S-GFP) GFP serving as the control group, diffusing uniformly throughout the cell. The findings revealed that ZmGF14–3 predominantly exhibited a punctate pattern of localization in protoplasts, primarily concentrating in the cytoplasm (**Figure 4B**).

### 3.6 Over-expression of ZmGF14–3 in *A. thaliana* and quantitation of GA, ABA, and IAA levels

**Figure 5:**
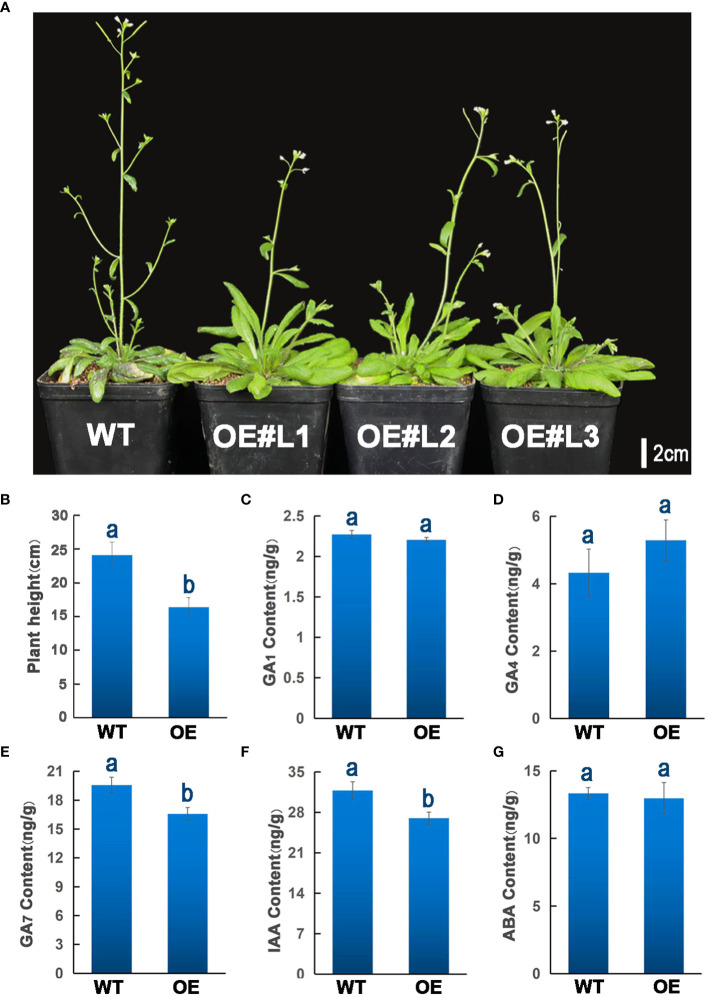
Over-expression of ZmGF14–3 in (A) *thaliana* and quantitation of GA, ABA, and IAA levels. **(A)** Over-Expression of ZmGF14–3 in (*A*) *thaliana*; **(B)** Statistical analysis of transgenic *Arabidopsis* and wild type plant height phenotypes; **(C–G)** Quantitation of GA_1_, GA_4_, GA_7_, IAA and ABA levels in transgenic *Arabidopsis* and wild type plant. The data represent means ± SD from three biological samples. Different letters indicate a significant difference between WT plants and different transgenic lines by Student’s t-test, * P < 0.05, the same letters indicate non-significant differences (P > 0.05).

To further explore the biological functions of ZmGF14–3, we heterologously expressed it in *Arabidopsis thaliana* for comprehensive functional research. This approach aims to elucidate the intricate roles and mechanisms associated with ZmGF14–3 in a different biological context, providing valuable insights into its potential functions. Each independent transgenic line underwent screening for hygromycin resistance. Subsequently, three distinct homozygous transgenic lines from the T3 generation (designated as OE#L1, OE#L2, and OE#L3), exhibiting the overexpression of ZmGF14–3, were meticulously chosen. At the flowering stage, we assessed the plant height of both the transgenic lines and the wild-type. Through rigorous statistical analysis, it became evident that transgenic plants manifested a considerably reduced height compared to their wild-type counterparts (*p* -Value < 0.05, as depicted in **Figures 5A, B**).

GF14s are essential genes that play a crucial role in regulating various aspects of plant growth and development, they are also involved in the metabolism of phytohormones, including GA, IAA and ABA, which are important for processes such as seed germination, root growth, and stress response (Qin et al., 2016). The phytohormone signaling pathway has undergone extensive exploration in *A. thaliana*, playing crucial roles in seed germination, stem elongation, flower transformation, and flowering. To investigate the impact of transgenic lines on phytohormone metabolism, we assessed the levels of GA, ABA, and IAA. The results unveil subtle distinctions in GA_1_, GA_4_, and ABA content between the overexpressing lines and the wild type (WT) (**Figures 5C, D, G**), while notably, a significant reduction in GA_7_ and IAA levels is evident in the overexpressing lines compared to WT (*p* < 0.05) (**Figures 5E, F**). This marked decline hints at the potential involvement of ZmGF14–3 in the signaling pathways of GA_7_ and IAA, thereby exerting a discernible influence on the developmental trajectory of plant height in the overexpressing *Arabidopsis*. These findings imply that ZmGF14–3 is putatively a key regulator in the phytohormone signaling pathway of *Arabidopsis*, thereby exerting an influence on the development of plant height.

### 3.7 The impact of ZmGF14–3 overexpression in *A. thaliana* on phytohormone responsE GENES

**Figure 6:**
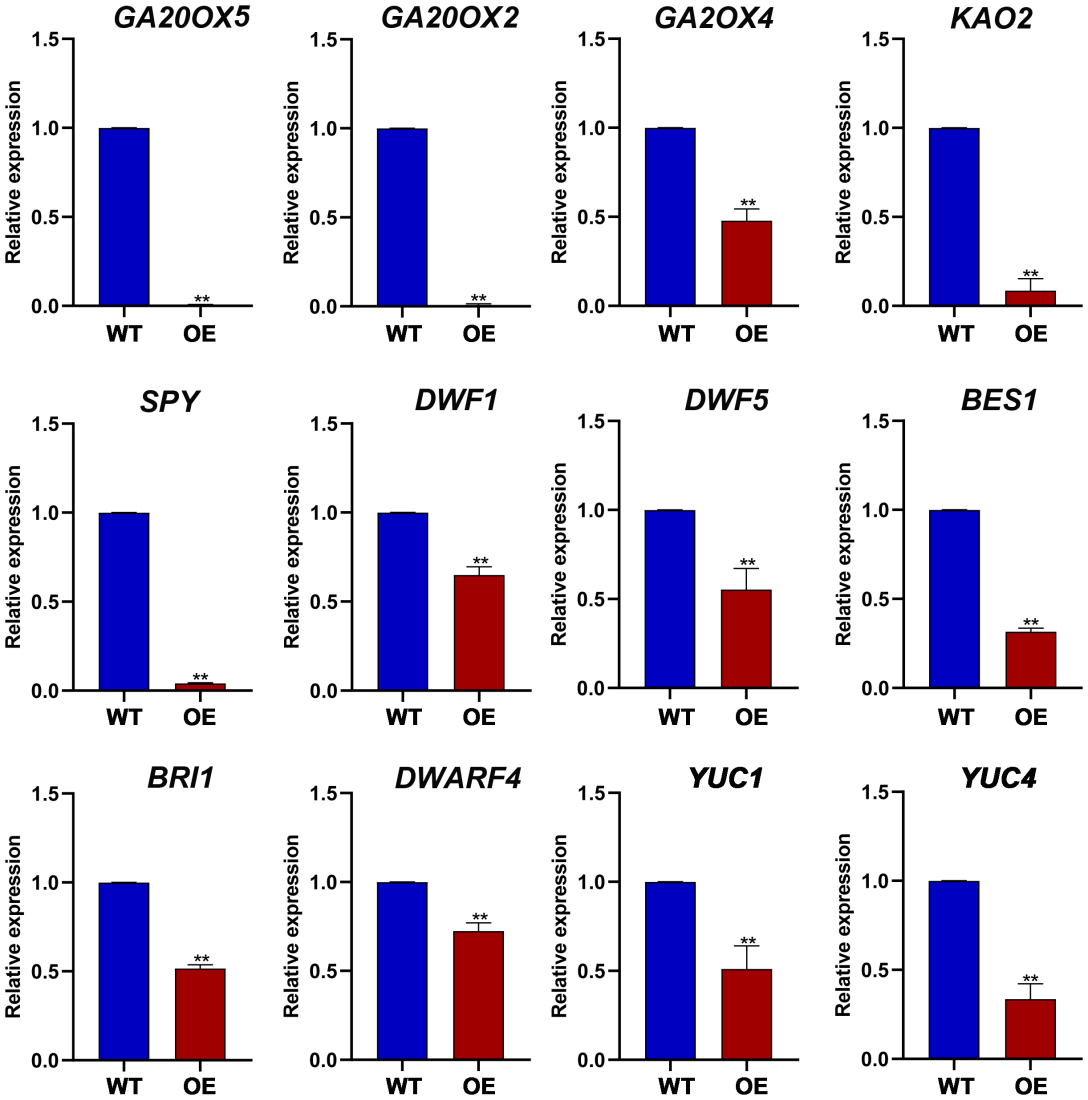
The expression levels of plant height related genes in transgenic *Arabidopsis* and wild type plants. ** indicates that the difference between OE and WT has reached a highly significant level (p-Value < 0.01).

To further investigate the impact of ZmGF14–3 overexpression on various endogenous phytohormone-responsive genes in *Arabidopsis*, we assessed the expression levels of genes associated with different phytohormones (**Figure 6**). For instance, within the gibberellic acid (GA) biosynthetic pathway, GA20ox2 plays a crucial role in converting GA_12_ to GA_9_, resulting in shortened internode length and pronounced dwarfism in the *ga20ox2* mutant of *Arabidopsis*. In *Arabidopsis* lines overexpressing ZmGF14–3, a notable decrease in the expression of GA20ox2 was observed, corresponding with the observed dwarf phenotype (Plackett et al., 2012). *DWF4* encodes a P450 protein responsible for catalyzing multiple 22a-hydroxylation reactions in the biosynthesis of BR. It serves as a key catalyst in a pivotal step that determines the overall flux in the biosynthetic pathway of BR, the *Arabidopsis dwf4* mutant exhibits a severely dwarfed phenotype accompanied by developmental abnormalities (Guo et al., 2010). In the biosynthetic pathway of plant growth phytohormones, the YUC gene family plays a pivotal role in regulating plant height and internode elongation. Research indicates that all YUC genes in *Arabidopsis* have overlapping functions, and the overexpression of each YUC gene results in excessive auxin production. Further studies demonstrate that the *Arabidopsis yuc1yuc4* double mutant exhibits pronounced dwarfing. In our investigation, the overexpression of ZmGF14–3 in *Arabidopsis* leads to a significant reduction in the expression levels of both *yuc1* and *yuc4* (Cheng et al., 2006). This result suggests that ZmGF14–3 may function as a key gene, capable of modulating the expression levels of associated genes within various phytohormones pathways, consequently impacting the developmental regulation of plant height.

## 4 Discussion

GF14s, serving as ubiquitous regulators, have been identified in a diverse array of plant species, including *Arabidopsis*, rice, wheat, soybean, cotton, rubber tree, alfalfa, and populus. This recognition began with the isolation of the first plant GF14 isoform from maize (Lu et al., 2022). The present study identified GF14s from 12 species, emphasizing the evolutionary patterns of ZmGF14s. Results found that in these GF14s, evolutionary pattern is conserved and could be identified in different surveyed eukaryotes, such as *mice*, *Micromonas pusilla*, and *Caenorhabditis elegans*, this suggests the ancient origin of GF14s. Generally speaking, conserved motifs and domains were generally regarded as important functional or regulatory elements (Zhu et al., 2022). In our results, 12 GF14s with similar conserved sequences were identified in a co-expression network associated with plant height in maize. Among them some key hub ZmGF14s exhibited co-expression with functionally validated genes associated with plant height, this results suggest that these genes may interact with plant height-related genes to regulate plant height development. Presently, GF14s exhibit interactions with a spectrum of genes related to energy metabolism, phytohormone signal transduction, redox homeostasis, stress response, and plant development. Recent studies have elucidated the additional involvement of GF14s and their binding proteins in the process of starch accumulation during cassava tuberization (Zhao et al., 2021). Currently, investigations into the GF14 gene family in plants are primarily focused on its pivotal role in regulating ion channels, hormone signaling pathways, and responses to diverse stresses, including but not limited to low temperature, salt, drought, osmotic stress, and mechanical stress (Gupta et al., 2023). While there is limited research on the regulation of plant height development by the GF14 gene family, sporadic literature reports suggest its potential key role in this aspect. Further investigations are warranted to deepen our understanding of the gene family’s influence on plant height development. As an illustration, the dephosphorylation of the shade-induced transcription factor PIF7 plays a crucial role in light signal perception and stem elongation. Mutations in the phosphorylation-resistant sites of PIF7 enhance its interaction with GF14 proteins, thereby facilitating the elongation of the hypocotyl stem. This study unveils a novel mechanism wherein GF14s regulate stem elongation in shaded conditions by sequestering PIF7 in the cytoplasm (Xu et al., 2018). In another research, it was demonstrated that the expression of GF14s in rice significantly influences internode elongation. This is attributed to the role of GF14, which encodes a signaling protein capable of binding and inhibiting the function of the RSG (repression of shoot growth) protein. The RSG protein acts as a transcriptional repressor of the kaurene oxidase (KO) gene in the gibberellic acid (GA) biosynthetic pathway (Phanchaisri et al., 2012). Therefore, stem elongation is a crucial agronomic trait that significantly influences plant height. Moreover, the increase in maize plant height primarily results from enhanced internode elongation rather than an increase in the number of internodes (Gao et al., 2023). To research the possible functions of ZmGF14s in plant height development, the mRNA levels of all 12 ZmGF14s in stems were surveyed in three hybrids at three different developmental stages, all ZmGF14s exhibited different expression patterns, from the results of phylogenetic relationships and the expression pattern of the ZmGF14s, we speculated regarding the possible functions of certain genes in certain developmental processes. During maize development, out of these ZmGF14s, the expression level of the ZmGF14–3 may demonstrate an increasing trend. Furthermore, it has been observed that this gene is notably expressed at high levels in the stem, this compelling evidence strongly suggests that ZmGF14–3 plays a pivotal role in orchestrating the process of stem elongation, thereby exerting a profound influence on the overall development of plant height. Thus, research on the growth and development of plant height in maize should primarily concentrate on ZmGF14–3, a specific gene of ZmGF14s.

Moreover, additional studies indicate that GF14 gene family can indirectly influence plant height development by regulating phytohormone metabolic pagibberellinthways. GF14 genes are crucial regulatory factors involved in multiple stress signaling pathways, participating in the signal transduction pathways of various phytohormone such as s, brassinosteroids, ABA, and ET (Zhao et al., 2021). For instance, In the GA synthesis pathway, a class of RSG transcription factors can regulate the expression of genes involved in gibberellin biosynthesis. Previous studies have shown that using RSG as bait protein can screen for target proteins of GF14 and identified the interacting site RSGSer114. Additionally, GF14 protein can acts as a negative regulatory factor by binding to RSG through phosphorylation of Ser-114 in RSG. Exogenous application of gibberellin can phosphorylate RSG, altering its cellular localization and preventing it from entering the nucleus to regulate the expression of target genes, thereby affecting gibberellin biosynthesis (Ito et al., 2014). *BZR1* and *BES1* play crucial roles as transcription factors in the brassinosteroid (BR) signaling pathway. When phosphorylated, BES1/BZR1 undergo structural changes that render them unstable and unable to bind to cis-elements, such as CPD, in downstream gene promoters. Instead, they are sequestered by GF14 proteins, leading to their degradation or retention in the cytoplasm (Nolan et al., 2017). GF14 proteins have the ability to recognize and interact with target proteins through specific amino acid sequences and phosphorylation forms. *OsBZR1*, *AtBZR1*, and *AtBES1* all possess a conserved GF14 binding domain. The study reveals that, via yeast two-hybrid experiments, five genes capable of interacting with *AtBZR1* were identified within the *Arabidopsis* GF14 family. In the case of rice, 8 GF14 proteins exhibiting interactions with OsBZR1 were screened. However, *BES1*/*BZR1* complexed with GF14 proteins are unable to translocate into the nucleus to regulate downstream gene expression, thereby hindering the transduction of BR signals and impacting plant height development (Lu et al., 2022). In *Arabidopsis*, some plant height-related genes involved in the GA pathway, such as *SPY*, *GA20ox4*, *KAO2*, *GA20ox2*, and *GA20ox5*, all exhibit significantly reduced expression levels in overexpression lines compared to the wild type (WT). After overexpression of ZmGF14–3 in *Arabidopsis*, genes involved in the Br metabolism pathway, such as *BES1*, *DWF5*, *DWF1*, *DWARF4*, and *BRI1*, as well as the IAA metabolism pathway gene *YUC1* and *YUC2*, all exhibited significantly reduced expression pattern. In summary, ZmGF14–3 may affect plant height development by regulating the expression levels of different phytohormone-related genes.

ZmGF14–3 demonstrated a co-expression relationship with pivotal genes associated with maize plant height, including *bv1*, *DWFI*, *ZmMADS3*, *BRI1*, *ZmCNR1*, *DWF4* and *ZmPGP1*. Among them, *bv1* plays a crucial role in auxin transport and exerts an influence on internode development. Maize mutants with *bv1* deficiencies exhibit a dwarf phenotype, underscoring the significance of *bv1* in the regulation of plant height (Avila et al., 2016). *DWF1* and *BRI1* play crucial roles in the signal transduction of brassinosteroids (BRs). The presence of mutations in *zmdwf1* and *bri1* genes resulted in a distinctive dwarfed phenotype. This highlights the significance of these genes in regulating plant growth and development through the BR signaling pathway (Jia et al., 2020). The *DWF4* gene encodes a C-22 hydroxylase, a pivotal enzyme in the brassinosteroid (BR) biosynthesis pathway. The overexpression of *ZmDWF4* in maize lines demonstrated a significant increase in BR levels, resulting in a remarkable enhancement in both grain yield and plant height (Liu et al., 2020). *ZmPGP1*, identified as an adenosine triphosphate (ATP) binding cassette (ABC) transporter, plays a pivotal role in facilitating polar auxin transport. Recent studies have compellingly demonstrated a significant correlation between the expression of *ZmPGP1* and the modulation of maize plant height, thereby emphasizing its key involvement in the intricate regulatory pathways governing plant growth (Li et al., 2019). As these genes have distinct functions in phytohormone metabolic pathways, it is plausible that ZmGF14–3 plays a crucial role in regulating the growth and development of maize by interacting with various phytohormone-related genes. Therefore, investigating the potential interactions between ZmGF14–3 and these genes could provide valuable insights into the molecular mechanisms underlying plant height development in maize. This exploration underscores the potential significance of GF14s in influencing pivotal factors for plant height, including cell elongation and internode development. Despite the current constraints in research, these insights lay the groundwork for delving into how GF14s contribute to overall plant growth, with a specific focus on height regulation. Subsequent inquiries in this domain hold the promise of revealing the nuanced roles of GF14s in shaping the architectural aspects of plants. This study is of great significance to the breeding of excellent maize varieties.

## Data availability statement

The datasets presented in this study can be found in online repositories. The names of the repository/repositories and accession number(s) can be found in the article/**Supplementary Material**.

## Author contributions

HW: Writing – original draft, Writing – review & editing, Funding acquisition. BW: Visualization, Writing – review & editing. LQ: Data curation, Writing – review & editing. YC: Formal analysis, Writing – review & editing. KC: Project administration, Writing – review & editing. DL: Software, Writing – review & editing. XS: Funding acquisition, Resources, Validation, Writing – review & editing. YZ: Data curation, Methodology, Writing – original draft. LL: Data curation, Funding acquisition, Project administration, Writing – review & editing.
