# Supplementary figures and images for: Deciphering the maize gene ZmGF14–3: implications for plant height based on co-expression networks
Source: Front Plant Sci. 2024 Jul 5;15:1397058. doi: 10.3389/fpls.2024.1397058 (PMC11257910; doi:10.3389/fpls.2024.1397058)

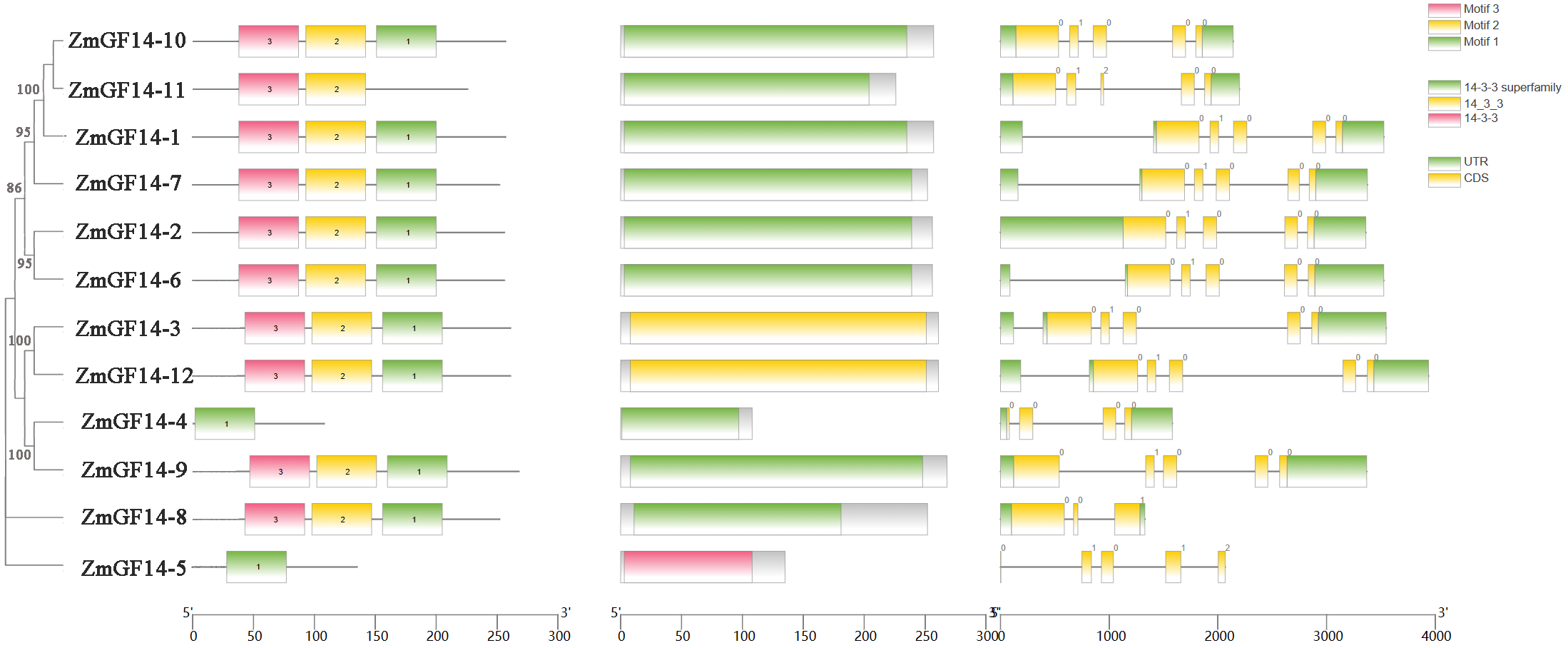

Supplement: Supplementary file 2 [file Image_1.tif]
