# Supplementary material for: Deciphering the maize gene ZmGF14–3: implications for plant height based on co-expression networks
Source: Front Plant Sci. 2024 Jul 5;15:1397058. doi: 10.3389/fpls.2024.1397058 (PMC11257910; doi:10.3389/fpls.2024.1397058)
Supplement: Supplementary file 3 [file Table_1.docx]

| Table S1 Fluorescence quantitative PCR primers for plant height-related genes in Arabidopsis | | | | |
| --- | --- | --- | --- | --- |
|  | Gene name | Gene ID | PCR primers | Involved phytohormone pathways |
| 1 | *GA20OX5*-F | AT1G44090 | TGTTGGCCCCCATAATGACC | GA-related |
|  | GA20OX5-R |  | AACGGTCTGCCAACTACCTG |  |
| 2 | GA20OX2-F | At5g51810 | TCTCTCAAGCCAAGACTCG |  |
|  | GA20OX2-R |  | AACTTTCCATCAAACGGTG |  |
| 3 | *GA2OX4*-F | AT1G60980 | TGTCCGACCAGTTAACCAGC |  |
|  | GA2OX4-R |  | GGCAGGGTCGTTAGTGTGAA |  |
| 4 | KAO2-F | At2g32440 | GTATGGGCGTACCGGGATTT |  |
|  | KAO2-R |  | AGAGCTTCAGGGCCATTGAC |  |
| 5 | SPY-F | AT3G11540 | AACTAGCCTGAAGCTGGCTG |  |
|  | SPY-R |  | CCATCTCCAAGTCACCACGG |  |
| 6 | DWF1-F | AT3G19820 | ACGAGATTGTTCTTGCGGGT | BR-related |
|  | DWF1-R |  | TCCAAGAGTTCCTTGCGACC |  |
| 7 | DWF5-F | AT1G50430 | GGGTCCAATATCTCCAGCCG |  |
|  | DWF5-R |  | TTCCAAACCACCAAAGACCA |  |
| 8 | BES1-F | AT1G19350 | TCCCGAGTCCTTCTCGAGTT |  |
|  | BES1-R |  | GACACTGGTGGAGTGACAGG |  |
| 9 | BRI1-F | AT4G39400 | TGAAACAGCACGCAAAACT |  |
|  | BRI1-R |  | TCATCCAAACACGCAACCG |  |
| 10 | DWARF4-F | At3g50660 | CGGTGATCTCAGCCGTACAT |  |
|  | DWARF4-R |  | TCCGTTGTTTTGCTGTTGCC |  |
| 11 | YUC1-F | AT4G32540 | TCATACTGCAAACAAAGTTG | IAA-related |
|  | YUC1-R |  | TCTCCTACAGACCACACCAA |  |
| 12 | *YUC4*-F | AT5G11320 | ATTTCTCCTACTTCTTGCTAA |  |
|  | YUC4-R |  | AAGTTATTTAGGATTATTAA |  |
| 13 | Actin-F | AT3G46520 | ATCTGCGAAAGGGTATCCAG | Reference gene |
|  | Actin-R |  | TGGAGAGCTTGATTTGCGAA |  |
