# Supplementary material for: Deciphering the maize gene ZmGF14–3: implications for plant height based on co-expression networks
Source: Front Plant Sci. 2024 Jul 5;15:1397058. doi: 10.3389/fpls.2024.1397058 (PMC11257910; doi:10.3389/fpls.2024.1397058)
Supplement: Supplementary file 4 [file Table_2.docx]

| Gene name | Gene identified | Exon number | CDS length(bp) | PI | MW(kDa) | Predicted location |
| --- | --- | --- | --- | --- | --- | --- |
| ZmGF14-1 | Zm00001d031688 | 6 | 774 | 4.81 | 29.00 | Chloroplast |
| ZmGF14-2 | Zm00001d032231 | 5 | 771 | 4.82 | 28.95 | Chloroplast |
| ZmGF14-3 | Zm00001d003401 | 6 | 786 | 4.76 | 29.6 | Chloroplast |
| ZmGF14-4 | Zm00001d007446 | 5 | 801 | 4.80 | 29.31 | Chloroplast |
| ZmGF14-5 | Zm00001d048868 | 7 | 747 | 5.27 | 27.9 | Cytoplasm |
| ZmGF14-6 | Zm00001d050375 | 6 | 771 | 4.78 | 28.86 | Chloroplast |
| ZmGF14-7 | Zm00001d052698 | 6 | 759 | 4.77 | 28.59 | Cytoplasm |
| ZmGF14-8 | Zm00001d052796 | 4 | 810 | 4.86 | 29.71 | Cytoplasm |
| ZmGF14-9 | Zm00001d053090 | 5 | 807 | 4.80 | 29.54 | Chloroplast |
| ZmGF14-10 | Zm00001d036226 | 5 | 774 | 4.76 | 29.03 | Cytoplasm |
| ZmGF14-11 | Zm00001d038649 | 5 | 774 | 4.81 | 29.07 | Cytoplasm |
| ZmGF14-12 | Zm00001d025617 | 6 | 786 | 4.75 | 29.63 | Chloroplast |

Table S2 Identification of ZmGF14s in co-expression network of plant height
