# Supplementary material for: Deciphering the maize gene ZmGF14–3: implications for plant height based on co-expression networks
Source: Front Plant Sci. 2024 Jul 5;15:1397058. doi: 10.3389/fpls.2024.1397058 (PMC11257910; doi:10.3389/fpls.2024.1397058)
Supplement: Supplementary file 7 [file Table_5.docx]

| Table S5 Genome information of plant species and outgroups | | | | |
| --- | --- | --- | --- | --- |
| Taxonomy | Species name | Abrr. | Genome Size（Mb） | GF14 number |
| Bromeliaceae | *Ananas comosus* | Ancom | 440.8 | 16 |
| Brassicaceae | *Arabidopsis thaliana* | AT | 119.7 | 14 |
| Nematode | *Caenorhabditis elegans* | Caele | 100 | 11 |
| Panicoideae | Maize | Zemay | 2191.6 | 12 |
| Rosaceae | *Malus domestica* | Madom | 647.511 | 46 |
| Mamiellaceae | *Micromonas pusilla* CCMP1545 | Mipus | 22 | 2 |
| Mammalia | Mus musculus | Muscu | 2662.4 | 7 |
| Poaceae | *Oryza sativa* | Orsat | 388.93 | 8 |
| Poaceae | *Setaria italica* | Seita | 441.705 | 8 |
| Solanaceae | *Solanum lycopersicum* | Lyesc | 805.374 | 13 |
| Solanaceae | *Solanum tuberosum* | Sotub | 810.123 | 12 |
| Andropogoneae | *Sorghum bicolor* | SoBic | 715.371 | 6 |
