# Supplementary material for: Deciphering the maize gene ZmGF14–3: implications for plant height based on co-expression networks
Source: Front Plant Sci. 2024 Jul 5;15:1397058. doi: 10.3389/fpls.2024.1397058 (PMC11257910; doi:10.3389/fpls.2024.1397058)
Supplement: Supplementary file 9 [file Table_7.docx]

Table S7 Gene expression data of ZmGF14s at three developmental stages (Lg(FPKM+1))

| Name | L1 | L2 | L3 | M1 | M2 | M3 | H1 | H2 | H3 |
| --- | --- | --- | --- | --- | --- | --- | --- | --- | --- |
| ZmGF14-1 | 1.53 | 1.64 | 1.89 | 1.49 | 1.5 | 1.8 | 1.61 | 1.27 | 1.87 |
| ZmGF14-2 | 2.28 | 2.28 | 2.44 | 2.21 | 2.13 | 2.38 | 2.26 | 2.15 | 2.36 |
| ZmGF14-3 | 2.44 | 2.57 | 2.69 | 2.46 | 2.51 | 2.7 | 2.44 | 2.54 | 2.64 |
| ZmGF14-4 | 1.88 | 1.77 | 1.78 | 2.08 | 1.98 | 2.03 | 1.82 | 1.81 | 1.74 |
| ZmGF14-5 | 1.07 | 0.83 | 1.33 | 1.07 | 0.92 | 1.06 | 1 | 0.83 | 1.04 |
| ZmGF14-6 | 2.12 | 2.08 | 2.41 | 2.09 | 2.05 | 2.49 | 2.21 | 1.95 | 2.57 |
| ZmGF14-7 | 1.24 | 1.33 | 1.65 | 1.18 | 1.23 | 1.67 | 1.41 | 1.2 | 1.68 |
| ZmGF14-8 | 1.8 | 2.1 | 1.77 | 1.81 | 2.12 | 1.84 | 1.82 | 2.22 | 1.89 |
| ZmGF14-9 | 2.17 | 2.21 | 2.3 | 2.17 | 2.09 | 2.21 | 2.18 | 2.11 | 2.08 |
| ZmGF14-10 | 0.97 | 0.74 | 1.24 | 1.01 | 0.86 | 1.44 | 0.9 | 0.84 | 1.22 |
| ZmGF14-11 | 0.11 | 0.14 | 0.2 | 0.19 | 1.2 | 1.21 | 0.29 | 0.37 | 0.34 |
| ZmGF14-12 | 2.22 | 2.5 | 2.47 | 2.4 | 2.63 | 2.62 | 2.4 | 2.55 | 2.57 |
